# Pacific plate motion change caused the Hawaiian-Emperor Bend

**DOI:** 10.1038/ncomms15660

**Published:** 2017-06-05

**Authors:** Trond H. Torsvik, Pavel V. Doubrovine, Bernhard Steinberger, Carmen Gaina, Wim Spakman, Mathew Domeier

**Affiliations:** 1 Centre for Earth Evolution and Dynamics (CEED), University of Oslo, 0315 Oslo, Norway; 2 Helmholtz Centre Potsdam, GFZ, Telegrafenberg, 14473 Potsdam, Germany; 3 NGU Geodynamics, Trondheim, Norway; 4 School of Geosciences, University of Witwatersrand, Johannesburg 2050, South Africa; 5 Department of Earth Sciences, University of Utrecht, 3584 Utrecht, The Netherlands

## Abstract

A conspicuous 60° bend of the Hawaiian-Emperor Chain in the north-western Pacific Ocean has variously been interpreted as the result of an abrupt Pacific plate motion change in the Eocene (∼47 Ma), a rapid southward drift of the Hawaiian hotspot before the formation of the bend, or a combination of these two causes. Palaeomagnetic data from the Emperor Seamounts prove ambiguous for constraining the Hawaiian hotspot drift, but mantle flow modelling suggests that the hotspot drifted 4–9° south between 80 and 47 Ma. Here we demonstrate that southward hotspot drift cannot be a sole or dominant mechanism for formation of the Hawaiian-Emperor Bend (HEB). While southward hotspot drift has resulted in more northerly positions of the Emperor Seamounts as they are observed today, formation of the HEB cannot be explained without invoking a prominent change in the direction of Pacific plate motion around 47 Ma.

## Abstract (web summary)

The Hawaiian-Emperor Chain has a 60° bend that has been interpreted as the result of Pacific plate motion at 47 Ma or drift of the Hawaiian hotspot. Here, the authors show that hotspot drift cannot be the dominant mechanism for bend formation, but involves a change in the direction of Pacific plate motion at ∼47 Ma.

The Hawaiian-Emperor Chain is one of the most spectacular geological features on Earth, stretching nearly 6,000 km from the active submarine volcano Lō'ihi near Hawaii to the Detroit (81–75 Myrs old) and Meiji (>82 Myrs old) seamounts in the northwest Pacific (Fig. 1). Wilson^1^ was the first to suggest that the Hawaiian Islands could be explained by the motion of the Pacific plate over a focused spot of melting in the mantle. The focused spot was later termed a ‘hotspot' by Morgan^2^ who proposed that hotspots are sourced by deep mantle plumes which remain fixed relative to each other and to the mantle. Morgan^3^,^4^ built the first kinematic model that defined absolute plate motions in a fixed hotspot reference frame, and in this model the Hawaiian-Emperor Bend (HEB) was attributed to a change in the absolute motion of the Pacific plate at the time of the bend formation. Many hotspot reference frames have subsequently been proposed, but explaining the HEB has proven problematic for frames assuming hotspot fixity. Pacific and Indo-Atlantic fixed hotspot reference frames do not agree with each other, and the motion of the Pacific plate reconstructed using Indo-Atlantic fixed hotspot reference frames fails to reproduce the geometry of the bend^5^,^6^,^7^,^8^,^9^. If the Pacific plate underwent a sudden directional change at ∼47 Ma—from a nearly northward direction (parallel to the Emperor Chain) to a north-westerly direction (corresponding to the Hawaiian Chain, Fig. 1)—attendant tectonic events along the margins of its neighbouring plates should be expected. According to Norton^6^, however, evidence for circum-Pacific tectonic events at the time of the bend formation is lacking, and hence the bend must rather reflect the motion of a non-stationary hotspot. In the decades since, this issue has remained unresolved, and continues to be vigorously debated^10^,^11^,^12^,^13^,^14^,^15^.

Palaeomagnetic data have proven helpful for testing the non-stationary hotspot hypothesis. If the volcanic chain was produced by a hotspot fixed in the mantle reference frame, and the rotation of the entire solid Earth (mantle and lithosphere) with respect to the spin axis (that is, true polar wander, TPW^16^) were negligible, then the palaeolatitudes inferred from palaeomagnetic analyses of volcanic rocks recovered from the Hawaiian-Emperor Seamounts should correspond to the present-day latitude of Hawaii (19.4°N). However, the palaeolatitudes of the Emperor Seamounts^17^,^18^,^19^,^20^,^21^,^22^ show a distinct trend of lower latitudes with decreasing age (Fig. 2). Detroit Seamount (∼80 Ma) yields a mean palaeomagnetic latitude ∼15° north of Hawaii, while the Palaeocene–Eocene seamounts (Suiko, Nintoku and Koko, ∼61–49 Ma) show northward offsets (relative to present-day Hawaii) of 8°–2°. Palaeomagnetic data from the Emperor Seamounts thus provide direct evidence for southward motion (possible longitudinal drift cannot be constrained from palaeomagnetic data) of the Hawaiian hotspot from Late Cretaceous to middle Eocene time (∼80–47 Ma)^20^,^21^,^22^, although this could be due to both motion of the hotspot and motion of the pole (TPW) with respect to the deep mantle. The idea that the hotspot was mobile during this time interval has been generally accepted by the geoscientific community, although with some dissenting voices^10^. Most geodynamic models also predict a drift of the hotspot to the south, and several recent studies argue that a rapid southward motion of the Hawaiian hotspot that ceased at ∼47 Ma can explain the formation of the HEB without requiring a significant change in the Pacific plate motion around that time^12^,^14^,^23^,^24^.

Here we discuss why the simple concept of a rapid southward motion of the Hawaiian hotspot is untenable to explain the HEB and show that, given the constraints on the motion of the Hawaiian hotspot from geodynamic modelling and palaeomagnetism, a change in the direction of Pacific plate motion around 47 Ma is a *conditio sine qua non* for explaining the formation of the HEB.

## Results

### Geometric considerations

**Figure 1:**
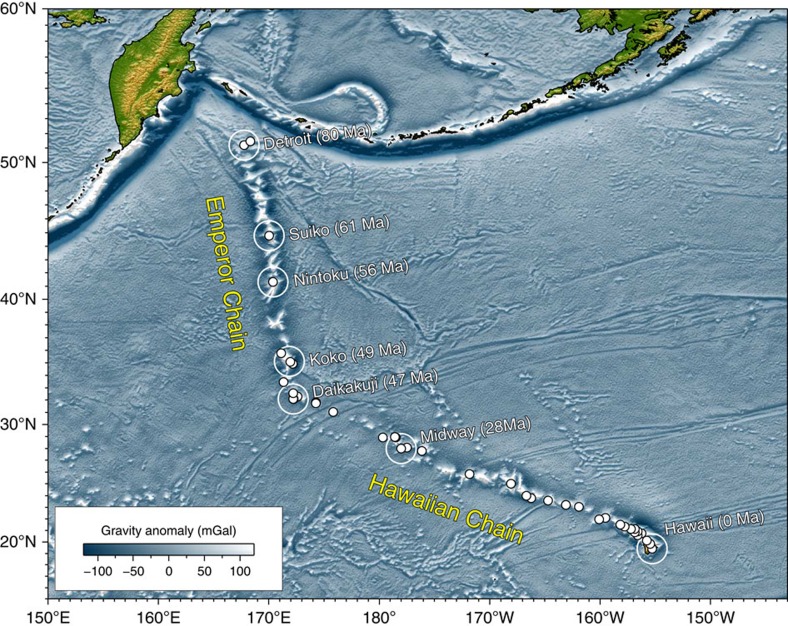
Hawaiian-Emperor Chain. White dots are the locations of radiometrically dated seamounts, atolls and islands, based on compilations of Doubrovine *et al*.^28^ and O'Connor *et al*.^40^. Features encircled with larger white circles are discussed in the text and Fig. 2. Marine gravity anomaly map is from Sandwell and Smith^48^.

Before discussing the estimates of Hawaiian hotspot drift available from geodynamic models and palaeomagnetic data, we find it instructive to provide an illustration of what kind of hotspot drift would be expected in the absence of a change in the Pacific plate motion at the time of the HEB. For this purpose, we adopt a simplified kinematic model, in which we assume that the Pacific plate moved with a constant angular velocity (*ω*=0.72°/Ma, about an Euler pole at 68°S, 103°E) from 80 Ma to the present. The location of the Euler pole and the rotation velocity were estimated so that the trend of the Hawaiian Chain (0–47 Ma) and the age of its oldest seamount (Daikakuji, ∼47 Ma, Fig. 1) are matched by the plate motion alone without any contribution from hotspot drift, i.e., assuming that the hotspot was stationary since 47 Ma (for example, ref. 10). The black line in Fig. 3a shows the model hotspot track that would be produced in this scenario of steady Pacific plate motion over a stationary hotspot since 80 Ma, and we attribute the discrepancy between its 80–47 Ma segment and the observed Emperor Chain geometry to the hotspot drift. For simplicity, we assume that the hotspot drifted with a constant angular velocity from 80 to 47 Ma and find that a combination of hotspot motion of ∼0.6°/Ma about an Euler pole at 44.3°S, 274.6°E (red line in Fig. 3a) and the motion of the Pacific plate reproduces the orientation of the Emperor Chain reasonably well, correctly placing the older end of the model track (yellow line in Fig. 3a) in the vicinity of Detroit Seamount (∼80 Ma).

This simple model highlights an important corollary of the Hawaiian-Emperor Chain geometry and age progression for the motion of the Hawaiian hotspot. Namely, if the Pacific plate moved steadily to the northwest before and after the bend formation, the hotspot drift must include a vast amount of westward motion over the 80–47 Ma period in order to satisfy the nearly north-south trend of the Emperor Chain^8^,^25^. In our exercise (Fig. 3a), the Hawaiian hotspot drifts ∼1,800 km to the west in addition to ∼1,000 km to the south, which is in good qualitative agreement with the estimates of hotspot drift inferred from kinematic models that do not feature a prominent change in the Pacific plate motion around the time of the bend^24^,^26^,^27^ (Fig. 4, see Methods). In stark contrast, all published estimates of hotspot drift based on geodynamic modelling (discussed in the next section) inevitably show the Hawaiian hotspot moving either from north to south (Fig. 5), or from NNW to SSE (for example, ref. 8, Model 4 in ref. 14). This is a consequence of Hawaii's location between the large upwelling under the Pacific (south of Hawaii) and regions of past subduction in the north Pacific^8^, which results in a relatively simple mantle flow geometry dominated by southward flow in the mid-to-lower mantle and northward flow in the uppermost part of the lower mantle (Fig. 6). There is therefore no geodynamic basis for a large westward component in the drift of the Hawaiian hotspot.

Next we show that geodynamic predictions of southward or SSE hotspot drift^8^,^14^,^28^ cannot be reconciled with the assumption that the motion of the Pacific plate did not change at the time of HEB formation. This is illustrated with another simple simulation (Fig. 3b) that considers a rather extreme scenario, in which we attribute the entire north-south length of the Emperor Chain (∼19°) to southward hotspot drift prior to the formation of the HEB at 47 Ma, and assume that the Pacific plate moved with the same angular velocity before and after the formation of the bend (as in Fig. 3a). In this scenario, the hotspot would have to move five times faster than the Pacific plate at a rate of 3.8°/Myr in order to reproduce the orientation of the Emperor Chain before the cessation of hotspot motion at 47 Ma (Fig. 3b). This extremely fast motion, however, implies that the entire Emperor Chain was created in just five million years, making the Detroit Seamount only 52 Myrs old (Fig. 3b), which is in clear contradiction with its radiometric age estimates (∼75–81 Ma). A better fit to the radiometric ages of the Emperor seamounts can be achieved by allowing the Pacific plate to move at a slower velocity in the same direction before the formation of the bend at 47 Ma (Fig. 3c), but this would require an extremely slow rate of ∼0.13°/Myr, followed by a nearly sixfold acceleration (to 0.72°/Myr) after 47 Ma, which is not supported by any observations or models.

Overall, our basic simulations (Fig. 3) and the insights from geodynamic modelling of the Hawaiian plume motion (Fig. 5) suggest that a change in the absolute motion of the Pacific plate is requisite for explaining the geometry and age progression of the Hawaiian-Emperor Chain. We now turn to absolute kinematic models that incorporate the geodynamic estimates of hotspot drift and their implications for Pacific plate motion.

### Moving hotspot reference frames

Steinberger *et al*.^8^ were the first to develop a self-consistent global model of absolute plate motions, mantle flow and resulting distortion of plume conduits and hotspot motion that was able to fit the geometries and age progressions of four hotspot tracks linked to the Hawaiian and Louisville hotspots in the Pacific, and to the Reunion and Tristan hotspots in the Indian and Atlantic oceans, respectively. Steinberger *et al*.^8^ initially attempted to reproduce the shape of the Hawaiian-Emperor Chain based on a classic plate circuit model linking the Pacific and Indo-Atlantic realms via Marie Byrd Land and East Antarctica (Model 1, Fig. 5b), but the calculated track plotted too much to the west of the Emperor Chain. An alternative plate circuit model (Model 2, Fig. 5b) linking Africa and the Pacific plate via Lord Howe Rise, Australia, and East Antarctica before 44 Ma improved the fit between the model track and the Emperor Chain. The fact that Model 2 gave an improved fit before 44 Ma does not necessarily mean that it is a perfect or best fit because the relative plate motion between Pacific and Lord Howe Rise in the Eocene is not that well known. Plate circuit Model 2 implies no relative motion between Pacific and Australia plates between cessation of spreading in the Tasman Sea and the transition to plate circuit Model 1, which may well be a simplification.

**Figure 2:**
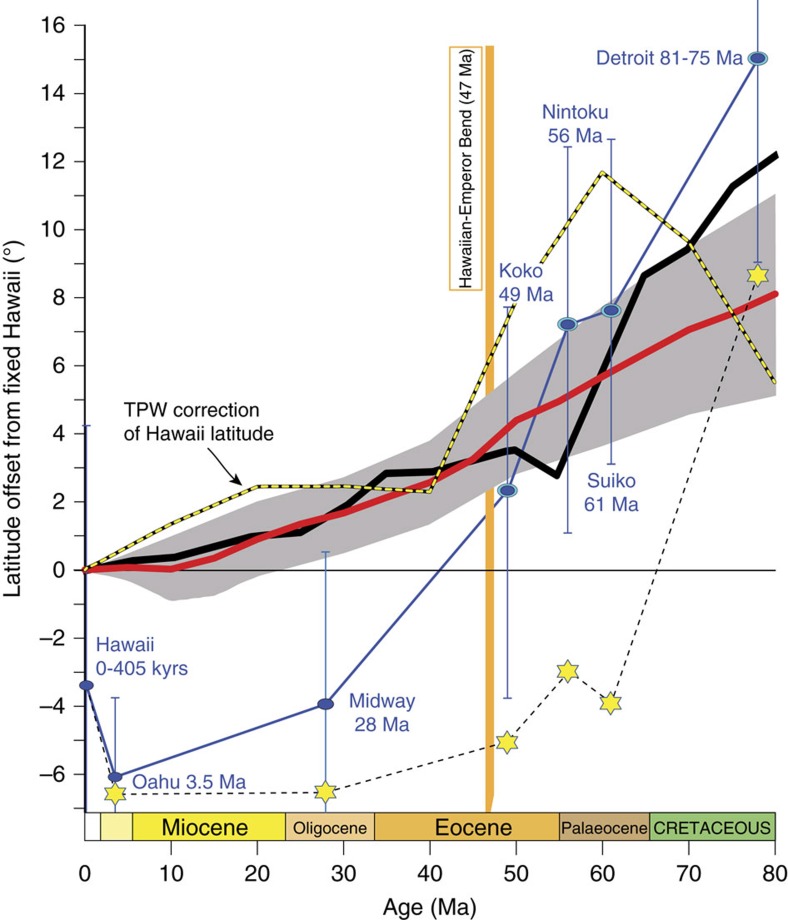
Latitudes and surface motion of the Hawaiian hotspot. Palaeomagnetically derived latitudes (blue ovals connected with the blue line) from seamounts along the Emperor Chain and islands/atolls along the Hawaiian Chain^17^,^18^,^19^,^20^,^21^,^22^,^49^,^50^ plotted with 95% confidence bars. The data are shown as latitude offsets from the present latitude of Hawaii (observed latitude minus latitude of Hawaii, 19.4°N). These offsets can reflect hotspot drift, the effect of TPW, non-dipole contributions to the time-averaged geomagnetic field in the vicinity of Hawaii (for example, the inclination anomaly corresponding to a ∼−3.5° latitude offset in the most recent data (<405 kyrs) from the Island of Hawaii^30^), or a combination of thereof (see text). The thick black line with yellow stippling shows TPW corrections for Hawaii (calculated from ref. 28, see Methods). TPW-corrected latitude offsets (except for Hawaii) are shown as six-pointed yellow stars connected with a dashed line; these were computed by subtracting the values of TPW correction from the observed palaeolatitudes. A newer palaeomagnetic result from Midway reported in abstract form^51^ gives a TPW-corrected offset of −3.2°. The palaeomagnetic results are compared with latitudinal estimates of surface motions of the Hawaiian hotspot (Fig. 5a,c) after Doubrovine *et al*.^28^ (thick red line with grey-shaded 95% confidence region) and Hassan *et al*.^14^ (thick black curve, their preferred model M3).

In a follow-up analysis that also included the New England Seamount Chain (Central Atlantic), Doubrovine *et al*.^28^ defined hotspot and absolute plate motions in a global moving hotspot reference frame that reproduced the Hawaiian-Emperor Chain well for the past 65 Myrs, and within reconstruction uncertainties for the past 80 Myrs (Fig. 5a). Remarkably, the trends of the Hawaiian and Emperor seamounts can be reproduced in this model even without the motion of the Hawaiian hotspot, so that the entire 60° bend is explained by a change in the Pacific plate motion (Fig. 7a,b). However, the model track that ignores the southward motion of the Hawaiian hotspot (red line in Fig. 5a) does not accurately match the Hawaiian-Emperor Chain. Although the orientation of its Hawaiian (∼0–50 Ma) and Emperor (∼50–80 Ma) segments are similar to those of the actual chain, the model hotspot track is shifted to the south of the Hawaiian Chain, and its north-trending Emperor segment is ∼800 km shorter than the Emperor Chain (Fig. 5a). These misfits reflect the necessity of a southward hotspot drift (∼8° between 80 and 10 Ma; red thick line in Fig. 2). Yet, in the model of Doubrovine *et al*.^28^ there is no noticeable change in the southward drift velocity at 47 Ma, and the formation of the sharp 60° bend is therefore chiefly a result of the directional change in the Pacific plate motion. A similar velocity of hotspot drift before and after the bend has also been advocated by Parés and Moore^29^ based on an entirely different data set.

While earlier models of plume motion^8^,^28^ were based on backward advection of present-day mantle density structures determined from tomographic models, Hassan *et al*.^14^ recently computed the surface motion of the Hawaiian hotspot using forward mantle convection modelling. Despite estimates of hotspot drift (Figs 2 and 5c) that are broadly similar to those of Doubrovine *et al*.^28^ (Figs 2 and 5a), they concluded that rapid southward motion of the Hawaiian hotspot before 47 Ma explains the formation of the bend without requiring a major plate motion change. However, the motion of the Pacific plate in the mantle frame model imposed by Hassan *et al*.^14^ also undergoes a prominent directional change at ∼50 Ma, and, similarly to the results of Doubrovine *et al*.^28^, the bend is reproduced by the combined plate and hotspot motions and mainly reflects the change in plate motion (red line in Fig. 5c).

### Palaeomagnetic estimates of hotspot drift and plate motion

The palaeolatitudes from the Emperor Seamounts (Detroit, Suiko, Nintoku and Koko) provide us with a direct record of the southward hotspot motion (∼0.42°/Ma) relative to the Earth's spin axis (Fig. 2), but translating the latitude offsets (15–2°) before the time of the bend into hotspot motion relative to the mantle requires corrections for TPW. Correcting palaeolatitudes for TPW reduces the latitude offsets and the corrected latitudes for the Palaeocene–Eocene seamounts plot below the latitude of Hawaii (Fig. 2). Only Detroit Seamount confidently yields a higher latitude estimate (∼9°) than that of Hawaii after TPW correction, and this latitude offset is in nearly perfect agreement with the geodynamic estimate of hotspot drift of Doubrovine *et al*.^28^. We note, however, that unlike the younger seamounts in the Emperor Chain, Detroit Seamount has a MORB (mid-ocean ridge basalt) geochemical signature, and as an alternative to plume advection, the latitude offset of the Detroit Seamount has also been suggested to result from ridge-plume interaction^23^.

Intuitively, it is perhaps peculiar that the TPW-corrected Palaeocene–Eocene seamount latitudes are lower than the latitude of the present-day Island of Hawaii. But in fact all reliable palaeomagnetic studies related to the younger Hawaiian Chain, including the Island of Hawaii itself (0–405 kyrs, ref. 30), report lower latitudes than the expected latitude of Hawaii, even without TPW corrections (Fig. 2). Explaining the latitude discrepancy of −3.4° for the young basalts from the Island of Hawaii^30^ by hotspot drift would require extremely fast northward motion of the hotspot over the past 0.4 Myrs, at a rate in excess of 8°/Myr, which is neither supported by modelling nor by the geometry of the chain. The Hawaiian hotspot overlies the north-eastern margin of the Pacific Large Low Shear-wave Velocity Province (LLSVP, Fig. 6a) in the lowermost mantle^31^,^32^, and the Hawaiian magnetic inclination (latitude) anomaly has been attributed to the strong lateral variations in core–mantle boundary heat-flow leading to non-dipole field contributions^30^. The proposed longevity of the Pacific LLSVP encompasses the Mesozoic and Cenozoic^32^ which suggests that this latitude anomaly—averaging to −5° for the past 3.5 Myrs—may have persisted over a longer time-scale and has similarly affected the palaeolatitude estimates from the older Emperor Seamounts. This poses a strong challenge for the inferences of hotspot drift derived from the palaeomagnetic latitude record of the Hawaiian-Emperor Seamounts because the lifetime of this anomaly and its possible variations in strength are unknown. This outstanding issue remains to be addressed.

The absolute kinematic model of Doubrovine *et al*.^28^ predicts dominantly northward movement of the Pacific plate—parallel to the Emperor Chain (Fig. 7a)—between 80 and 47 Ma, and attributes only ∼4° of the total north-south length of the Emperor Chain (∼19°) to southward hotspot drift. Palaeomagnetic data from localities on the Pacific plate unrelated to the Hawaiian-Emperor Chain can be combined to define a Pacific apparent polar wander (APW) path which, when corrected for TPW, can give us an independent estimate of the absolute amount of northward plate motion during formation of the Emperor Chain. There are several published Pacific APW paths (for example, refs 25, 33), but the temporal resolution of these paths is limited, and they largely rely on palaeomagnetic poles derived from a mixture of sediment and basalt core palaeolatitudes, modelling of seamount magnetic anomalies (inversion of magnetic and bathymetric data) and the analysis of the magnetic anomaly skewness (ref. 25). Pacific APW paths should therefore be used with caution, but our revised APW path (Fig. 8; Table 1), when corrected for TPW, indeed predicts a northward shift of the Pacific plate (blue line in Fig. 7c) that is almost identical to that estimated from the absolute kinematic model^28^, supporting its general veracity.

## Discussion

**Figure 3:**
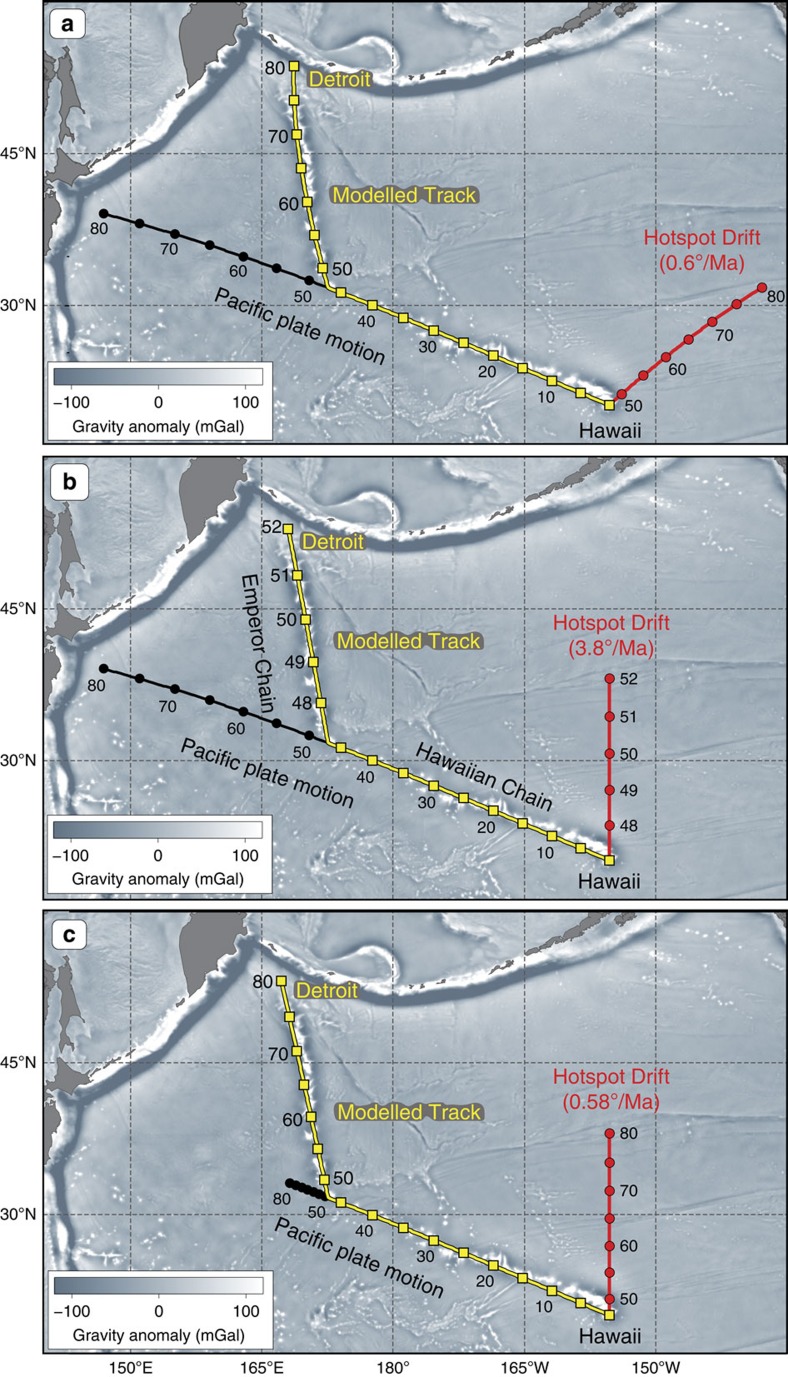
Simulating the Hawaiian-Emperor Bend. (**a**) A simulation that assumes that the Pacific plate moves with a constant angular velocity to the north-west (*ω*=0.72°/Ma, Euler pole: 68°S, 103°E, see text), which requires south-westward hotspot motion (by 20.1° about the Euler pole at 44.3°S, 274.6°E, ∼0.6°/Ma) in order to form the Emperor Seamounts during the 80–47 Ma period and reproduce the geometry of the Hawaiian-Emperor Chain. (**b**) To produce a 60° bend with the Pacific plate not changing its direction and velocity (same as in plate a) and purely southward hotspot drift (∼19° of motion corresponding to the north-south extent of the chain) requires an extreme rate of hotspot motion (3.8°/Ma), which would imply that the Emperor Chain has been created in just 5 Myrs and Detroit Seamount is only 52 Myrs old. (**c**) To form the Emperor Chain over the 80–47 Ma period with the southward hotspot drift of ∼19° of motion, (∼0.58°/Ma) and no change in the direction of plate motion we must assume that the Pacific plate has moved at an extremely slow rate before 47 Ma (0.13°/Myr), which was followed by a nearly sixfold velocity increase after the formation of the bend (to 0.72°/Ma).

**Figure 4:**
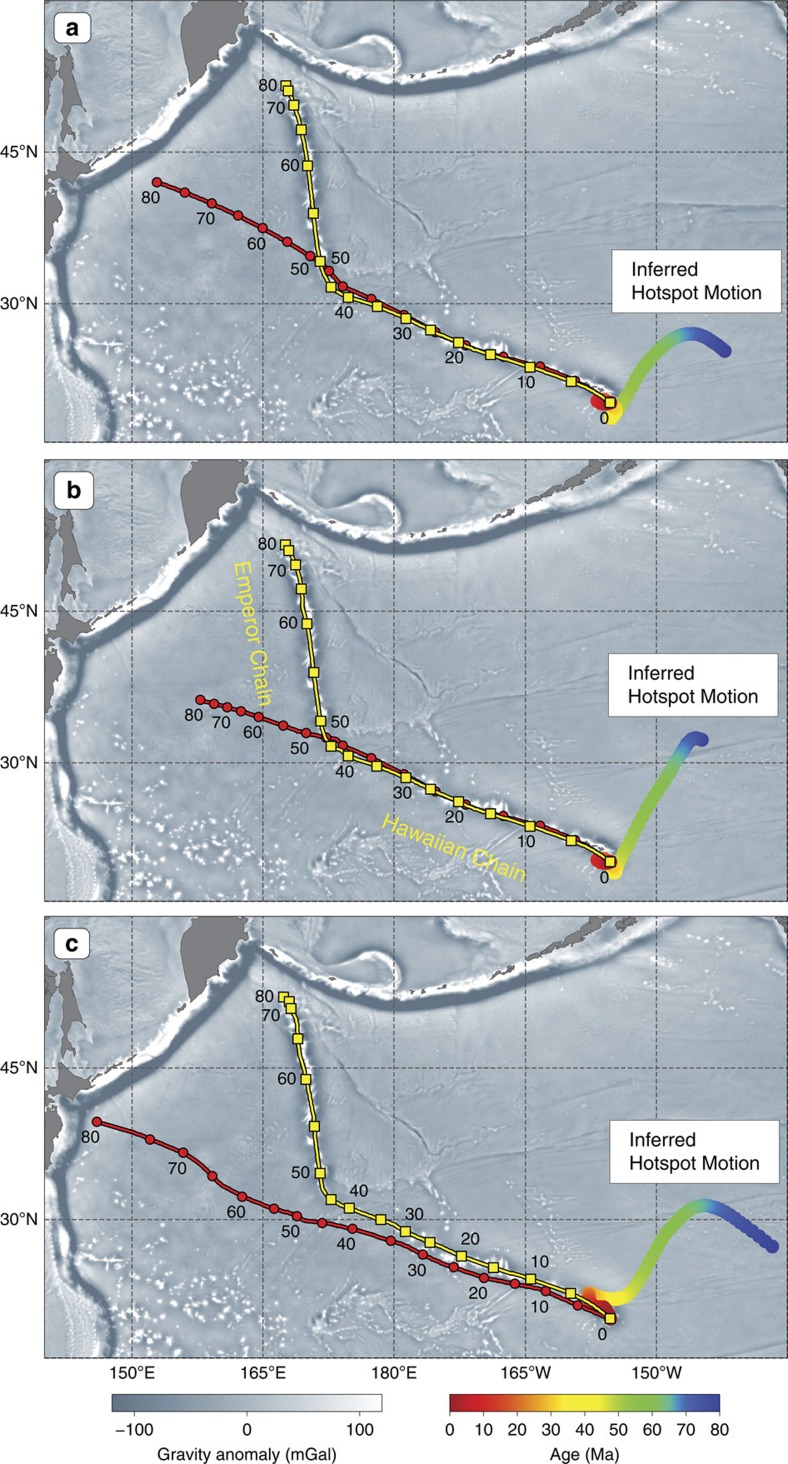
Motion of the Hawaiian hotspot inferred from absolute kinematic models. The multi-coloured swaths represent inferred paths of hotspot drift, which, when combined with the Pacific absolute plate motion (APM), produce model hotspot tracks (yellow lines) that accurately track the geometry of and the age progression along the Hawaiian-Emperor Chain (see Methods for details). The red lines track the Pacific APM over an assumed fixed hotspot. Three APM models that do not feature a prominent change in the Pacific plate motion at the time of the HEB formation were considered. (**a**) Pacific APM model of Chandler *et al*.^27^ (WK08-D). (**b**) The geodynamic APM model of Butterworth *et al*.^24^ (42–72 Myr ago), augmented by the rotations from Wessel and Kroenke^52^ (WK08-A) for the younger ages, and extrapolated to 83 Ma as in Wessel & Müller^53^. (**c**) APM model based on the Indo-Atlantic moving hotspot reference frame of O'Neill *et al*.^39^ and using the relative plate motions of Seton *et al*.^26^ updated by Shephard *et al*.^54^

**Figure 5:**
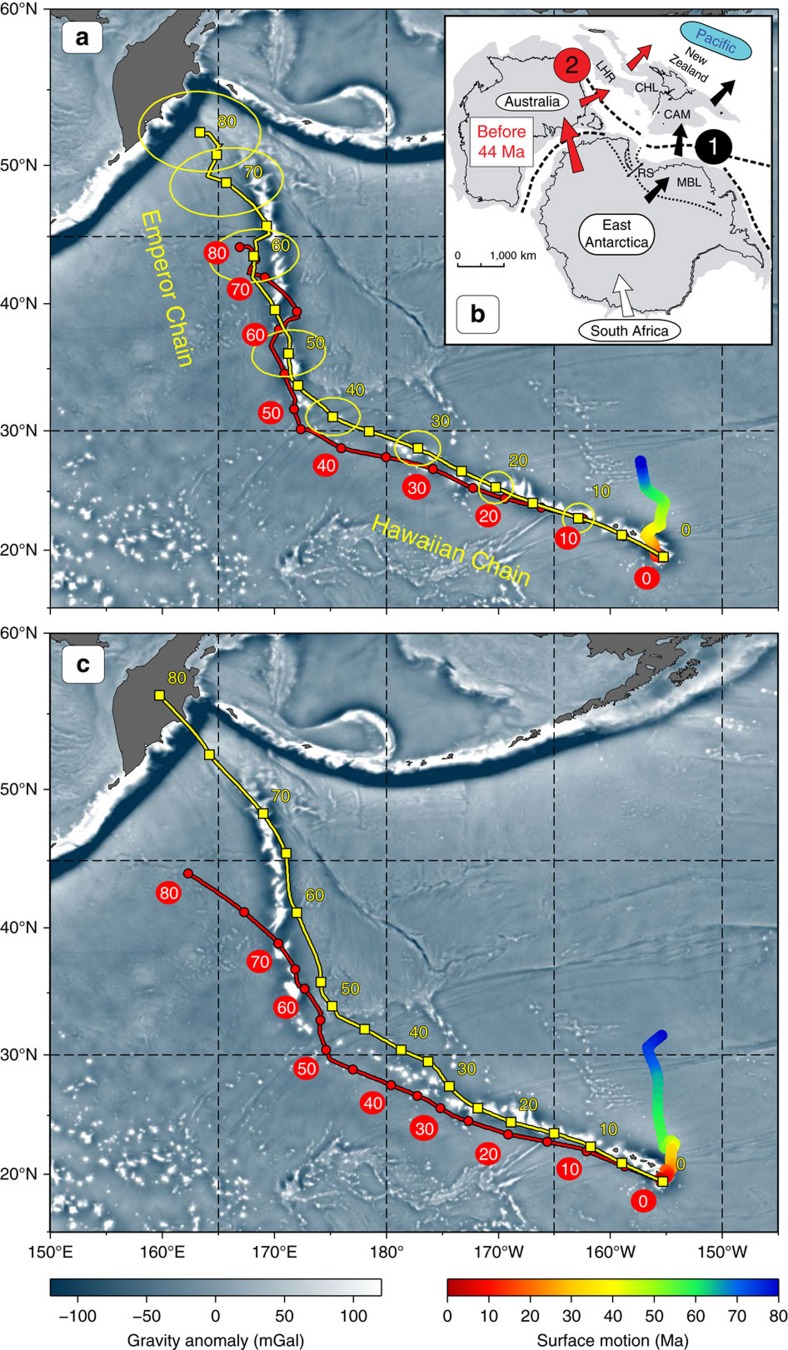
Models of Hawaiian hotspot track and hotspot surface motion. (**a**) Model hotspot track (yellow thick line; yellow squares with uncertainty ellipses at 10 Myr intervals) computed by combining the motion of the Pacific plate in the global moving hotspot reference frame^28^ (using Model 2 plate circuits of panel **b**) and the geodynamically modelled surface motion of the Hawaiian hotspot (rainbow-coloured swath; see ref. 28 for details). The red line shows the track that would be produced if the Hawaiian hotspot were fixed in the same reference frame, that is, reflects the plate motion alone. The difference between the yellow and red lines corresponds to the surface hotspot motion. (**b**) Two relative plate circuit models used for reconstructing relative motions between the Pacific plate and plates of the Indo-Atlantic hemisphere (for example, Africa). After chron 20 time (43.8 Ma), Models 1 and 2 follow the same plate motion chain through East Antarctica and Marie Byrd Land^8^. LHR, Lord Howe rise; CHL, Challenger plateau; CAM, Campbell plateau; MBL, Marie Byrd Land; RS, Ross Sea. (**c**) Model tracks of the moving and fixed Hawaiian hotspot (yellow and red lines, respectively) calculated using the plate motion model of Hassan *et al*.^14^, which is based on the global moving hotspot reference frame of Torsvik *et al*.^55^ but uses modified relative plate motions^56^. Similarly to **a**, the track for the moving hotspot (yellow line) combines the motion of the Pacific plate (traced by the red line) and the surface hotspot motion (rainbow-colored swath) from the preferred geodynamic model of ref. 14 (model M3). Because the hotspot drifts to the south in both models (plates **a**,**c**), the bends in the modelled tracks (yellow lines) reflect the directional change of the Pacific motion at ∼45–50 Ma, whereas the southward hotspot drift contributes to produce a longer track, which is shifted to the north relative to the track that would be produced by a fixed hotspot (red lines).

**Figure 6:**
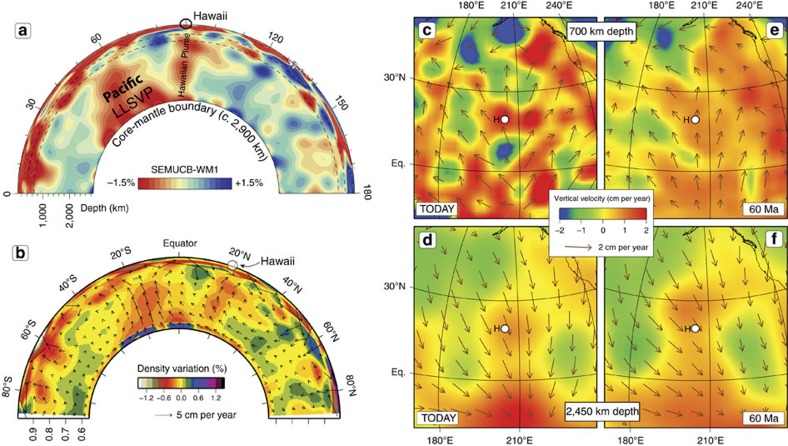
Imaging the Hawaiian Plume and geodynamic modelling. (**a**) North-south vertical slice of the SEMUCB-WM1^43^ mantle tomography model through the Pacific LLSVP (Large Low Shear-wave Velocity Province^31^,^32^) and the Hawaiian Plume. Axis numbers indicate arc degrees from the start of the profile. (**b**) Cross-section from the South Pole to the North Pole at 155°W and map views at 700 and 2,450 km depth, at times 0 Ma (today) and 60 Ma (**c**–**f**). The mantle flow geometry (see Methods) is dominated by southward flow in the mid-to-lower mantle (**d**,**f**) and northward flow in the uppermost part of the lower mantle (**c**,**e**) and in the upper mantle. White circles indicate location of Hawaii (H).

**Figure 7:**
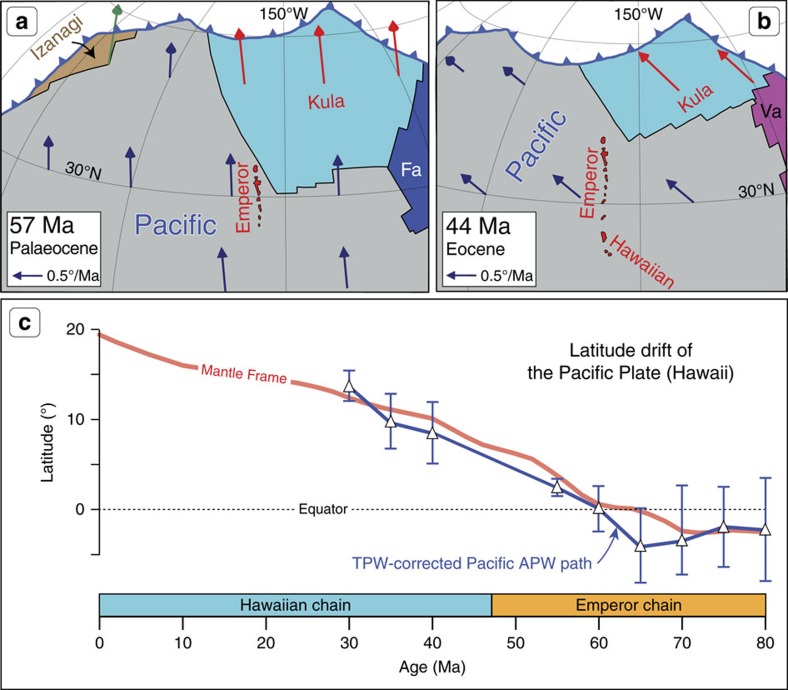
Plate reconstructions and latitudes. (**a**,**b**) Absolute North Pacific plate reconstructions and plate velocity vectors^28^ before and after the time of the Hawaiian-Emperor bend. The Kula plate formed at ∼83 Ma from pieces of the Izanagi, Farallon (Fa), and Pacific plates^26^,^57^, and was subducted at ∼40 Ma. The Izanagi plate was subducted at ∼55 Ma, while the Vancouver (Va) plate formed at ∼52 Ma. (**c**) Latitudinal motion of a location on the Pacific plate, which is presently at the position of Hawaii (19.4°N, 155.2°W), calculated using the Doubrovine *et al*.^28^ model of the Pacific absolute plate motion (red line labelled ‘Mantle Frame') and the Pacific APW path (Fig. 8b) corrected for TPW (white triangles with 95% confidence bars, connected with blue lines; see Methods and Table 1).

The Pacific plate is the largest plate on Earth—covering ∼20% of its surface for the past 80 Myrs—and it is therefore crucial to understand the Pacific plate motion history for building robust global plate kinematic and geodynamic models. Simple considerations of the geometry and age progression of the Hawaiian-Emperor Chain (Fig. 3) show that it would be extremely difficult—if possible at all—to reproduce the HEB if no change in the Pacific plate motion occurred at about 47 Ma. Kinematic models without a significant change in Pacific plate motion around the time of the bend formation necessitate a large westward component in the total hotspot drift (Fig. 4) that is not supported by geodynamic models (Fig. 5). The southward hotspot drift that is predicted by these numerical models lacks a significant component of westward motion, reflecting simple flow geometry beneath the northern Pacific region, which is governed by the persistent large-scale upwelling above the Pacific LLSVP (Fig. 6) and subduction of oceanic slabs at the northern rim of the Pacific Ocean (Fig. 7a).

**Figure 8:**
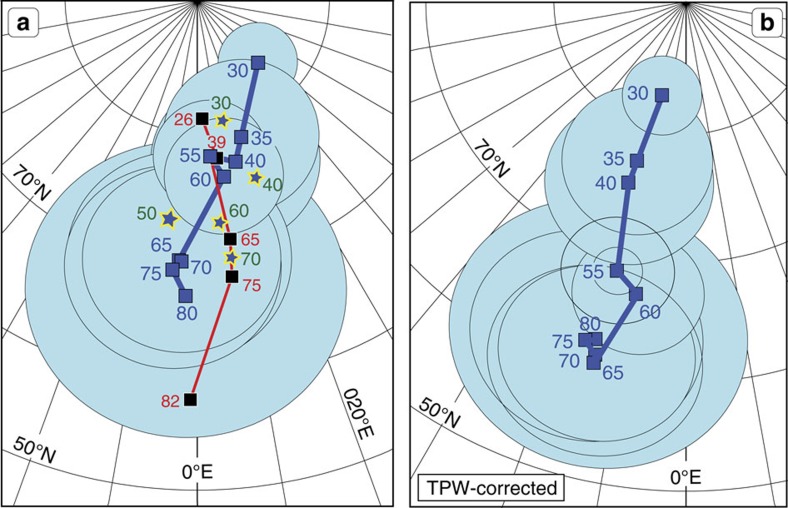
Pacific apparent polar wander. (**a**) A revised APW path for the Pacific plate (Table 1) is shown by blue squares with a blue line and A_95_ ovals (light blue shading). Black squares connected with red lines is a Pacific APW path by Gordon and Van der Voo^33^. Blue/yellow star symbols are Pacific running mean poles (time window poles, all data types) listed in Beaman *et al*.^25^. Both data sets are shown without confidence ovals for clarity. (**b**) TPW-corrected Pacific path (see Methods). Numbers show the ages Myrs±5 Myr (that is, computed with a 10 Myr running mean). See Table 1 for details.

The hotspot drift predicted by geodynamic models amounts to ∼4–9° (Fig. 2) of dominantly southward motion between 80 and 47 Ma (at a rate of ∼0.2–0.4°/Ma), which is insufficient to produce the ∼19° length of the Emperor Chain if no change in the direction of the Pacific plate motion has occurred. Attributing the formation of the bend solely to the southward hotspot drift not only requires higher rates of hotspot motion (∼0.6°/Ma), but also implies unrealistically slow motion of the Pacific plate during the 80–47 Ma interval followed by a nearly sixfold acceleration after 47 Ma (Fig. 3c). This is geodynamically implausible. In contrast, kinematic models in which the Pacific plate motion undergoes a directional change at ∼47 Ma (Fig. 5) are capable of reproducing the geometry and ages of the Hawaiian-Emperor Chain with the moderate amounts of southward hotspot drift quoted above. While the southward hotspot drift helps in lengthening the Emperor Chain compared to what would be observed if the Hawaiian hotspot were not moving, the change from the nearly northward orientation of the Emperor Chain to the north-westward trend of the Hawaiian Chain at ∼47 Ma manifested by the 60° bend is chiefly a result of the change in the direction of the Pacific plate motion observed in these models (Fig. 7a,b). A strong component of northward motion of the Pacific plate during the formation of the Emperor Seamounts (Fig. 7c) suggested by the Pacific APW path (Fig. 8b) provides independent evidence in support of this scenario.

The interpretation of the palaeomagnetically derived latitudes of the Emperor Seamounts (Fig. 2) in terms of hotspot drift in the mantle reference frame depends on corrections for TPW, which are not trivial or unique since TPW rotations always rely on absolute kinematic models with which they are defined, and different absolute plate models predict different TPW corrections (for example, ref. 28) and hence different histories of latitudinal motion for the Hawaiian hotspot. The possibility of a persistent local magnetic inclination (latitude) anomaly in the vicinity of the Hawaiian hotspot arising from non-dipole fields further complicates inferences of hotspot drift derived from direct palaeomagnetic estimates. Given these limitations, great caution should be exercised when taking the palaeomagnetic estimates from the Emperor Seamounts at face value as a record of the southward hotspot drift with respect to the mantle.

After more than two decades debating hotspot drift versus Pacific plate motion change to explain the HEB, we must realize that neither of these two end-member options is able to accurately reproduce the geometry and age progression of the Hawaiian-Emperor Chain. While the change in the direction of the Pacific plate motion is required to account for the geometry of the bend, the more than 2,000-km-long stretch of the Emperor Seamounts would not have been created had the Hawaiian hotspot not drifted southward from Late Cretaceous to middle Eocene time (Fig. 5). If we accept that, we can stop going in circles and move forward, focusing new research on understanding the processes that resulted in the change in the direction of the Pacific plate motion at around 47 Ma, which we conclude is a prerequisite for explaining the formation of the HEB. The directional change at ∼47 Ma demands plate reorganizations and tectonic events in the Pacific realm, but the causes and mechanism(s) for these events are still unknown. Many of the Early Cenozoic components of the Pacific (Fig. 7a,b) have since been subducted, accreted or otherwise modified, and the former plate geometries are often speculative. Nevertheless, the continental margins along the Pacific still carry piecemeal geologic evidence that along with seismic tomography^34^,^35^,^36^ can be used to improve existing reconstructions, and ultimately answer the question of what drove the Pacific plate motion to change at ∼47 Ma.

## Methods

### Palaeomagnetic data and TPW corrections

We only analysed palaeomagnetic data collected from Emperor Seamounts and Hawaiian islands/atolls that are exclusively derived from volcanic rocks linked to the Hawaiian hotspot. We do not include palaeomagnetically derived latitudes in Fig. 2 from sediments (prone to inclination shallowing effects) or those derived from seamount magnetic anomalies near the bend^37^. TPW-corrected latitudes for Hawaii (Fig. 2) were determined by a direct comparison of the most up-to-date global palaeomagnetic^38^ and moving hotspot^28^ reference frames. The yellow-dashed curve for TPW corrections in Fig. 2 is the angular distance between the location of Hawaii (155.3°W, 19.4°N) and the north pole (70.6°) minus the angular distance between Hawaii and the (northern hemispheric) TPW path of Doubrovine *et al*.^28^ at the corresponding age. For example, if, due to TPW, the north pole was displaced towards Hawaii, the second distance would be smaller than 70.6°, and the difference would be positive. Accordingly, the TPW-corrected latitude curve (yellow stars in Fig. 2) was computed by subtracting the values of TPW correction (read from the yellow-dashed curve) from the observed palaeolatitudes (blue ellipses). This procedure is not strictly correct given that the TPW corrections were computed for a constant hotspot position. More precise estimates could be obtained in an iterative manner, but this would only lead to minor modifications, trivial in comparison with the uncertainties of the palaeolatitude data.

The Pacific APW path in Fig. 8a was calculated from poles based on the analysis of magnetic anomaly skewness and modelling of magnetic anomalies associated with seamounts. We excluded data from the Hawaiian-Emperor Seamounts because of possible non-dipole field contributions. To correct the APW path for TPW we rotated the Pacific palaeomagnetic poles to the South African frame (Model 2 plate circuits, Fig. 5b), corrected the rotated poles for the motion of Africa in the global moving hotspot frame^28^ (that is, transferred them into the mantle frame), rotated the corrected poles back to the Pacific co-ordinates, and calculated a new running mean path (Fig. 8b; Table 1).

### Inferences of hotspot motion from absolute plate kinematics

The estimates of hotspot drift shown in Fig. 4 were obtained by reconstructing the eruption sites of radiometrically dated seamounts of the Hawaiian-Emperor Chain (Fig. 1) using finite rotations describing the motions of the Pacific plate in three absolute reference frames^24^,^27^,^39^ that do not feature prominent directional changes of the Pacific plate motion at the time of the formation of the HEB (47 Ma); see the caption of Fig. 4 for the sources for these reference frames. The locations of dated seamounts at their respective ages^28^,^40^ (Fig. 1) were reconstructed using GPlates software^41^. The reconstructed locations were approximated by a spherical spline with a smoothing parameter of 20 (ref. 42) to define an age progression for the inferred hotspot motion at 1 Ma increments. The low value of the smoothing parameter (20) was selected to ensure that the inferred hotspot motions were reasonably smooth, but at the same time, when combined with the absolute motions of the Pacific plate, they would accurately predict the geometry of the Hawaiian-Emperor Chain and the age progression of the dated seamounts. The spline-approximated models of the inferred hotspot motion are shown as multi-coloured swaths in Fig. 4.

As a sanity check we computed model hotspot tracks (yellow lines in Fig. 4) by combining the hotspot and plate motions as described in Doubrovine *et al*.^28^. Because the hotspot motion was computed using the absolute plate kinematics, the model tracks in all experiments are essentially forced to replicate the actual geometry and age progression of the Hawaiian-Emperor Chain almost exactly; the departures arising from spline smoothing are small (on the order of few tens of kilometres) and are not significant for the purpose of our analysis.

### Geodynamic modelling

The density model shown in Fig. 6 is computed from SEMUCB-WM1 (ref. 43) (panel a) with the method described in Steinberger^44^. To consider compositionally distinct LLSVPs we added a density anomaly of 1.2% wherever in the lowermost 300 km of the mantle the shear-wave anomaly is less than −1%. Otherwise, the model is constructed as in the reference model of that paper^44^. The viscosity structure used to compute the flow is Model 2b (the preferred model) of Steinberger and Calderwood^45^ and phase boundaries are treated as in Steinberger^46^. Surface plate motions are from Torsvik *et al*.^47^, which have a net rotation, but that is gradually reduced to zero with depth.

The mantle flow model that was used to compute hotspot motion in Fig. 5a is similar to the one shown in Fig. 6, but is based on an earlier mantle tomography model. Different from earlier models, SEMUCB-WM1 (ref. 43) appears to resolve the Hawaiian plume, and the predicted mantle flow field features an upwelling beneath Hawaii, separated from the large-scale upwelling above the Pacific LLSVP by a downwelling that appears as a blue or green blob in Fig. 6b,c. Such a separate upwelling is not predicted with earlier tomography models, where Hawaii rather occurs in the middle of a large-scale convection cell, and predicted Hawaii hotspot motion is comparatively large. In contrast, only a few degrees of motion are predicted if the flow model of Fig. 6 is used.

### Data availability

The authors declare that all relevant data are available within the article. Other pertinent data are available from the authors upon request.

## Additional information

**How to cite this article:** Torsvik, T. H. *et al*. Pacific plate motion change caused the Hawaiian-Emperor Bend. *Nat. Commun.* **8,** 15660 doi: 10.1038/ncomms15660 (2017).

**Publisher's note**: Springer Nature remains neutral with regard to jurisdictional claims in published maps and institutional affiliations.

## Supplementary Material

Peer Review File

## Figures and Tables

**Table 1:** Pacific apparent polar wander path.

**Age (Ma)**	**Pacific APW path**				**TPW-corrected**			
	***N***	**A**_**95**_	**Plat**	**Plon**	**A**_**95**_	**Plat**	**Plon**	**Lat. Hawaii**
30	1	∼3.0	83.5	44.6	∼3.0	82.7	345.6	N13.7 ^+1.8^/_−1.7_
35	4	5.9	79.2	17.9	5.7	77.3	342.9	N 9.8 ^+3.2^/_−3.0_
40	3	6.4	77.5	13.4	6.4	75.5	342.4	N 8.5 ^+3.5^/_−3.3_
55	1	∼1.9	78.2	4.8	∼1.9	68.6	345.7	N 2.5 ^+1.0^/_−1.0_
60	3	4.5	76.5	8.7	5.0	67.1	350.6	N 0.2 ^+2.5^/_−2.5_
65	5	8.5	70.0	356.0	8.2	60.6	345.7	S 4.0 ^+4.2^/_−4.1_
70	6	7.5	70.0	356.5	7.5	61.3	345.7	S 3.4 ^+3.9^/_−3.8_
75	5	8.2	69.2	354.7	8.9	62.4	343.4	S 1.9 ^+4.5^/_−4.5_
80	3	12.2	67.2	357.7	11.4	62.6	345.2	S 2.2 ^+5.8^/_−5.7_

APW, apparent polar wander; TPW, true polar wander.

The Pacific APW path (Fig. 8) was calculated from magnetic anomaly skewness and palaeomagnetic modelling of seamounts^58^,^59^,^60^,^61^,^62^,^63^,^64^,^65^,^66^. We excluded all data from the Hawaiian-Emperor Seamounts because of possible non-dipole field contributions as discussed in the text. The APW path is a running mean path (10 Myr window lengths) based on 15 palaeomagnetic input poles (32–82 Ma). *N*=number of input poles; A_95_=95% confidence circle; Plat/Plon=Pole latitude/longitude. TPW corrections are based on Doubrovine *et al*.^28^ (see Methods). We also list the palaeomagnetically reconstructed latitude (Lat.) for Hawaii (19.4°N) after TPW corrections (Fig. 7c). Note that mean-poles for 30 and 55 Ma are based on only one pole and therefore A_95_ is approximated from the original poles (32 and 57 Ma, respectively^59^,^62^), where 95% confidence limits are reported as major and minor semi-axis lengths.
