# Supplementary material for: Pacific plate motion change caused the Hawaiian-Emperor Bend
Source: Nat Commun. 2017 Jun 5;8:15660. doi: 10.1038/ncomms15660 (PMC5465363; doi:10.1038/ncomms15660)
Supplement: Peer Review File [file ncomms15660-s1.pdf]

Reviewers' Comments:

Reviewer #1 (Remarks to the Author)

First off - this manuscript addresses a question (the origin of the bend in the Hawaiian - Emperor seamount chain) that is fundamental to our understanding of the plate tectonic and paleogeographic evolution of the entire Pacific realm through the past 100 million years. Determining whether the bend reflects a change in the motion of the Pacific plate, or if it is simply a function of the behaviour of the hotspot responsible for the seamount chain has far-reaching implications, impacting how we interpret the orogenic belts that surround the Pacific; our view of mantle tomographic images of subducted slabs globally; and the links between geological events (e.g. collision of India and Asia) and the global plate response. Indeed, our ability to confidently reconstruct Earth's paleogeography and distribution of plates and plate boundaries back into the Precambrian is very much in doubt if we can't resolve the Eocene movement of Earth's largest plate. So this is a paper that very much deserves to be published in Nature; it will find a broad audience spanning everything from climate modelers, through paleomagnetists, and mantle dynamicists.

The paper itself is well written, clear and concise. I have attached a marked up copy of the manuscript with a small number of suggested revisions, but the paper could be published as is. Similarly, the story laid out by the authors is straightforward. Simple plate and hotspot motion models are used to test the predictions of various end-member scenarios for explaining the bend: almost all the result of plate motion versus almost all the result of hotspot motion. The predictions of these models strongly suggest that a significant change in plate motion is required to explain the bend. The question then is just how much of a change in plate motion is required. To address this question the authors have to utilize simple models of mantle dynamics and constrain the amount of true polar wander. Again, the authors employ the principal of least astonishment throughout the development of their arguments, and the result is a well-constrained estimate of both the amount of hotspot motion and the change in the Pacific plate trajectory.

Figure 2 is the meat of the paper, and it could be better labeled. For example, a fair number of readers are going to spend an inordinate amount of time wondering why present day Hawaii plots at -3.3 degrees from the Hawaiian reference line. Figure 2 is first referred to on line 51, but the explanation (the Hawaiian inclination anomaly) isn't explained until line 173. APW paths are typically referenced to a specific site (inset map on Fig. 2) but unless I missed it, I couldn't figure out or find the reference site on any of the figures. And the caption for figure 4 is strangely regurgitated in the manuscript (lines 280 - 286).

Perhaps the weakest part of the paper is the lack of any treatment of the geological data from the circum-Pacific. As the others point out, much of the debate over the nature of the Hawaiian - Emperor bend stems from the 1995 paper by Ian Norton. The authors write (line 41) "According to Norton, however, evidence for circum-Pacific tectonic events at the time of the bend formation is lacking, and hence the bend must rather reflect the motion of a non-stationary hotspot." The authors never come back to this point and hence there remains some confusion: if the authors are correct and the bend requires a significant change in the motion of the Pacific plate at 47 Ma, then why is there no evidence for circum-Pacific tectonic events at that time? Addressing this question is almost certainly outside of the scope of this paper (and would require a significant amount of additional text), but at the very least the authors could point out this apparent conundrum: making this point could prompt a significant amount of new research.

Recommendation: Accept

Stephen T. Johnston

Reviewer #2 (Remarks to the Author)

I enjoyed this paper, I am sure will be a "bending point" in the field of the so-called unifying theory in Earth Sciences. See my specific comments below.

Conventional wisdom is that a combination of absolute plate motion and hot spot drift are both necessary to explain the Emperor-Hawaiian seamount chain bend. In the submitted manuscript, Trond Torsvik and colleagues go beyond and postulate that the Hawaiian-Emperor bend (HEB) requires not only plate motion change and drift of the Hawaiian hotspot, but further, that the direction of the Pacific Plate motion must have changed dramatically at ca 47 Ma (the "knee" of the bend), in addition to a moving Hawaiian hotspot. With elegance (nice figures by the way) and solid arguments, albeit sometimes hard to follow due to the nature of the problem, the authors furnish data to support that in addition to a moving hotspot frame until 47 Ma, the Pacific Plate motion experienced a pronounced change in the drift direction at that time.
A prerequisite of their story is what Doubrovine et al. (2012) already coined as the "global moving hotspot reference frame" (or GMHRF) for estimating hot spot motions. In fact, the paper under revision, in my opinion, is in a way a corollary of Doubrovine et al. Notice the number of times that the concept of "true polar wander (TPW) correction" appears within the text. Nothing wrong with it, but something that perhaps needs to be highlighted as it stays a bit shadowed in the present form. The first reference to TPW is found in Figure 2, as an inset. Still, as the authors state (line 162), the latitude offset of the Detroit Seamount is "in nearly perfect agreement with the geodynamic estimate of hotspot drift …" after the TPW correction.
I gather that both (a) a cease (or a decrease?) of the hotspot (although in contradiction to previous observations as stated above, - an issue that need to be at least addressed) and (b) a pronounced change in drift direction of the Pacific Plate, are required to occur at around 47 Myr- what is the putative cause for such coincident major plate/mantle event?
Finally, and beyond the scope of this neat paper, it remains an enigmatic question which is the cause of such dramatic change in mantle convection (?), or plate kinematic scenario that seemingly occurred at around 47 Ma. Really worth to pursue and I am sure it will stimulate lots of speculation in the 'geodynamic community'.
Some specific comments follow below:
Line 51: On Figure 2. I think that using latitude as the sole proof for drift is a bit misleading …. What if the entire movement was along a parallel? In other words, the "longitudinal" component of drift is not reflected on the lines plotted in the diagram. So please consider and/or comment on that.
Line 59: It is correct that most recent models imply a cease of the Hawaiian hotspot motion at 47 Ma. Still, I recall that a Michigan team (I believe Moore and Pares?, published in EPSL, 2005- reference is missing in the submitted paper) determined that (1) the Hawaiian hotpot movement as inferred from the study of the high biogenic productivity belt at the Equator, and (2) the Hawaiian hotspot moved at about 0.3deg/My to the SE since 43 Myr, whereas the Pacific Plate drift north at ca 0.2deg/Myr. Such rates are rather slow, but faster that those inferred from model of Figure 3b. So, perhaps to assert that "no observations … support such extremely slow motion …" (line 112) might be somewhat "arguable".
Line 170: Could such lower than expected latitudes be a hint of a rapid and recent drift to the north? Please provide some further explanation.

Line 157: the 0.4deg/Ma is the same order of magnitude as the one obtained using the productivity zone approach (Moore-Pares paper mentioned above), I think this is encouraging.

Figure 2: Why not to describe the inset first (as this is the first prominent data that one sees), and then the X-Y plot? This is a key figure. Also, does it really help including the horizontal black line labeled "Hawaii Fixed"? I think it does not! Note that the Y-axis reads "… fixed Hawaii". Please consider deleting this line for simplicity, or else include in the caption the purpose of such line. Also I would refer to "six-pointed star" instead of David star (just an opinion).

Figure 3: "… angular velocity to the NW".

Otherwise this is a great contribution to the Earth Sciences community, and specifically to plate tectonics and beyond, and deserves to be published after considering the points brought up in this review.

Reviewer #3 (Remarks to the Author)

This is a valuable and timely review paper addressing an important issue: the extent to which the HEB is due to a change in the motion of the Pacific plate versus a change in the southward drift of the Hawaiian Hotspot. For a while it was generally accepted that both changes in Pacific plate motion and in the drift of the Hawaiian Hotspot were involved in the formation of the HEB. But several recent papers, e.g. by Wright et al. (2015) in Geology and Hassan et al. (2016) in Nature, have argued that the Bend is primarily due to changes in the drift of the Hawaiian Hotspot and that changes in Pacific plate motion were not important. I thought that Wilson's (2016) reply in Geology to Wright et al. (2015) really hit the nail on the head; he showed that there was indeed a very significant change in Pacific-Antarctic motion at 47 Ma at the latitude of the Hawaiian hotspot which Wright et a. (2015) had overlooked. I think that figure 5 from the paper under review here also nails the importance of Pacific Absolute plate motion changes: take out Hassan et al.'s southward drift of the hotspot (Figure 5c) and you still have the Bend. Q.E.D.

This is not to say that this paper is fast reading. There are so many complex issues involved, that unless you are up on all of the various topics, parts of the paper are difficult to follow. The authors might want to consider moving more of the material to the Methods section. For example, it might be sufficient just to keep figure 6a in the paper and move the rest of figure 6 to the Methods section.

The title of this paper gets it right: the HEB is a mind-bending mystery. So, just to add to the list of mind-bending problems, I will point out that one issue that is given short shrift in this paper are the uncertainties in the plate circuit connecting the Indo-Atlantic plates to the Pacific plate. Because of the poorly constrained motion between East and West Antarctica prior to Chron 20, most studies these days looking at global plate motions, including this one, use the circuit from Ant-Aus-LHR-Pac first proposed by Steinberger et al. (2004). This gives an "improved fit" when linking Africa to Pacific hotspot trails. Great paper. However, the fact that this circuit gives an "improved fit" doesn't mean that it is the correct fit. In fact the plate motions between the LHR and the Pacific plate in the Eocene are not that well known. If the authors really take the LHR to be fixed to the Pacific plate between the cessation of spreading in the Tasman Sea at Chron 24 and the jump to using the East-West Antarctic-Pacific link at Chron 20 then that constrains there to be no motion between the Australia and Pacific plates in this time interval, i.e. making it one huge plate. Is that realistic? It would help to have some statement from the authors, perhaps after line 129, addressing the "unknown uncertainties" in the plate circuit and whether they might have any effect on the HEB mystery.

Reviewed by Steve Cande

A couple of typos:

Line 115: "a of change" should be "a change"
Line 146" "Despite" shou

Response to Reviewers' comments:

Reviewer #1 (Remarks to the Author):

First off - this manuscript addresses a question (the origin of the bend in the Hawaiian - Emperor seamount chain) that is fundamental to our understanding of the plate tectonic and paleogeographic evolution of the entire Pacific realm through the past 100 million years. Determining whether the bend reflects a change in the motion of the Pacific plate, or if it is simply a function of the behaviour of the hotspot responsible for the seamount chain has far-reaching implications, impacting how we interpret the orogenic belts that surround the Pacific; our view of mantle tomographic images of subducted slabs globally; and the links between geological events (e.g. collision of India and Asia) and the global plate response. Indeed, our ability to confidently reconstruct Earth's paleogeography and distribution of plates and plate boundaries back into the Precambrian is very much in doubt if we can't resolve the Eocene movement of Earth's largest plate. So this is a paper that very much deserves to be published in Nature; it will find a broad audience spanning everything from climate modellers, through paleomagnetists, and mantle dynamists.

The paper itself is well written, clear and concise. I have attached a marked up copy of the manuscript with a small number of suggested revisions, but the paper could be published as is.

**RESPONSE:** Suggested manuscript revisions have been implemented in the revised text
Similarly, the story laid out by the authors is straightforward. Simple plate and hotspot motion models are used to test the predictions of various end-member scenarios for explaining the bend: almost all the result of plate motion versus almost all the result of hotspot motion. The predictions of these models strongly suggest that a significant change in plate motion is required to explain the bend. The question then is just how much of a change in plate motion is required. To address this question the authors have to utilize simple models of mantle dynamics and constrain the amount of true polar wander. Again, the authors employ the principal of least astonishment throughout the development of their arguments, and the result is a well-constrained estimate of both the amount of hotspot motion and the change in the Pacific plate trajectory.<br><br>Figure 2 is the meat of the paper, and it could be better labelled. For example, a fair number of readers are going to spend an inordinate amount of time wondering why present day Hawaii plots at -3.3 degrees from the Hawaiian reference line. Figure 2 is first referred to on line 51, but the explanation (the Hawaiian inclination anomaly) isn't explained until line 173.
**RESPONSE:** We have clarified the origin of the Hawaiian latitude offset in the revised figure caption. We have also removed the inset APW paths to simplify the diagram and moved the original supplementary Fig. 1 to the main article body (new Figure 8). Thus, the inset map in Fig.2 is no longer required.
APW paths are typically referenced to a specific site (inset map on Fig. 2) but unless I missed it, I couldn't figure out or find the reference site on any of the figures.
**RESPONSE:** APW paths are referenced to a specific plate (i.e. the Pacific in our case) but not to a specific site. Reference to a specific site, however, is required to calculate latitude drift (site location is Hawaii in Figure 7c).
And the caption for figure 4 is strangely regurgitated in the manuscript (lines 280 - 286).
**RESPONSE:** We have removed lines 280-286 in methods since already in Figure 4 caption
Perhaps the weakest part of the paper is the lack of any treatment of the geological data from the circum-Pacific. As the others point out, much of the debate over the nature of the Hawaiian - Emperor bend stems from the 1995 paper by Ian Norton. The authors write (line 41) "According to Norton, however, evidence for circum-Pacific tectonic events at the time of the bend formation is lacking, and hence the bend must rather reflect the motion of a non-stationary hotspot." The authors never come back to this point and hence there remains some confusion: if the authors are correct and the bend requires a significant change in the motion of the Pacific plate at 47 Ma, then why is there no evidence for circum-Pacific tectonic events at that time?<br><br>Addressing this question is almost certainly outside of the scope of this paper (and would require a significant amount of additional text), **but at the very least the authors could point out this apparent conundrum: making this point could prompt a significant amount of new research.**
**RESPONSE:** In this paper we deliberately avoided to discuss geological data from the circum-Pacific that could be linked to plate reorganization at 47 Ma, and do not speculate on the geodynamic mechanisms that may have led to this event. As recognized by the reviewer, addressing these questions would require an extensive and detailed review of geological evidence from circum-Pacific regions, which is outside the scope of our analysis and will be the focus of future research. Therefore, we have opted for pointing out the issue in broad, general terms, stating in the last paragraph of the "Discussion" that "The directional change at ~47 Ma *demands* plate

reorganizations and tectonic events in the Pacific realm, but the causes and mechanism(s) for these events are still unknown," and "the continental margins along the Pacific still carry piecemeal geologic evidence that along with seismic tomography[34-36] can be used to improve existing reconstructions, and ultimately answer the question of what drove the Pacific plate motion to change at ~47 Ma." We think that these two statements clearly express the question raised by the reviewer, and our paper will provide a stimulus for further research aimed at solving this apparent conundrum.

Recommendation: Accept
Stephen T. Johnston

Reviewer #2 (Remarks to the Author):

I enjoyed this paper, I am sure will be a "bending point" in the field of the so-called unifying theory in Earth Sciences. See my specific comments below.
Conventional wisdom is that a combination of absolute plate motion and hot spot drift are both necessary to explain the Emperor-Hawaiian seamount chain bend. In the submitted manuscript, Trond Torsvik and colleagues go beyond and postulate that the Hawaiian-Emperor bend (HEB) requires not only plate motion change and drift of the Hawaiian hotspot, but further, that the direction of the Pacific Plate motion must have changed dramatically at ca 47 Ma (the "knee" of the bend), in addition to a moving Hawaiian hotspot. With elegance (nice figures by the way) and solid arguments, albeit sometimes hard to follow due to the nature of the problem, the authors furnish data to support that in addition to a moving hotspot frame until 47 Ma, the Pacific Plate motion experienced a pronounced change in the drift direction at that time.

A prerequisite of their story is what Doubrovine et al. (2012) already coined as the "global moving hotspot reference frame" (or GMHRF) for estimating hot spot motions. In fact, the paper under revision, in my opinion, is in a way a corollary of Doubrovine et al. Notice the number of times that the concept of "true polar wander (TPW) correction" appears within the text. Nothing wrong with it, but something that perhaps needs to be highlighted as it stays a bit shadowed in the present form. The first reference to TPW is found in Figure 2, as an inset. Still, as the authors state (line 162), the latitude offset of the Detroit Seamount is "in nearly perfect agreement with the geodynamic estimate of hotspot drift …" after the TPW correction.

RESPONSE: We have added one sub-clause in the introduction to highlight the importance of TPW a bit more (Lines 78-79).

I gather that both (a) a cease (or a decrease?) of the hotspot (although in contradiction to previous observations as stated above, - an issue that need to be at least addressed) and (b) a pronounced change in drift direction of the Pacific Plate, are required to occur at around 47 Myr- what is the putative cause for such coincident major plate/mantle event?

RESPONSE: This is not entirely correct. As we discussed in the paper, while it would be not possible to produce the Hawaiian-Emperor bend without a prominent directional change in the Pacific plate motion at 47 Ma, geodynamic models yield contrasting predictions for the changes in the rate of hotspot drift at this time. Some models do show a decrease in hotspot drift after 47 Ma (e.g.,

Hassan et al., 2016), whereas others (in fact, the majority of models) do not feature a prominent change in the drift rate (e.g., Steinberger et al., 2004; Doubrovine et al., 2012). Because it is not clear whether a prominent slowdown of the hotspot drift occurred or not at the time of the bend formation, we think it is premature to speculate on causes of the "coincident" changes in plate motion and hotspot drift at around 47 Ma.

Finally, **and beyond the scope of this neat paper**, it remains an enigmatic question which is the cause of such dramatic change in mantle convection (?), or plate kinematic scenario that seemingly occurred at around 47 Ma. Really worth to pursue and I am sure it will stimulate lots of speculation in the 'geodynamic community'.

RESPONSE: See response to reviewer 1 above

<u>Some specific comments follow below:</u>

Line 51: On Figure 2. I think that using latitude as the sole proof for drift is a bit misleading …. What if the entire movement was along a parallel? In other words, the "longitudinal" component of drift is not reflected on the lines plotted in the diagram. So please consider and/or comment on that.

RESPONSE: We have added that longitudinal drift is unconstrained from the palaeomagnetic data in the introduction (Line 56).

Line 59: It is correct that most recent models imply a cease of the Hawaiian hotspot motion at 47 Ma. Still, I recall that a Michigan team (I believe Moore and Pares?, published in EPSL, 2005-reference is missing in the submitted paper) determined that (1) the Hawaiian hotpot movement as inferred from the study of the high biogenic productivity belt at the Equator, and (2) the Hawaiian hotspot moved at about 0.3deg/My to the SE since 43 Myr, whereas the Pacific Plate drift north at ca 0.2deg/Myr. Such rates are rather slow, but faster that those inferred from model of Figure 3b. So, perhaps to assert that "no observations … support such extremely slow motion …" (**line 112**) might be somewhat "arguable".

RESPONSE: We think that the reviewer misinterprets the estimate of Parés and Moore (2005). First of all, "the Pacific drift north at ca 0.2 deg/Myr" quoted by the reviewer (25 mm/yr in the original paper) refers only to the northward component of the Pacific plate motion averaged over the last 53 Myrs. To arrive at the total plate velocity over the 0-43 Ma period, Parés and Moore (2005) supplemented this northward rate with the estimate of east-west plate velocity of ca. 59 mm/yr based on recent GPS measurements – which in itself is a questionable approach because there is no reason to expect that this instantaneous rate is an accurate estimate of the average longitudinal velocity over the entire time interval. But even if we accept this value, the total plate velocity would be ca. sqrt(25**2+59**2) = 64 mm/yr or ca. 0.6 deg/Myr. This is much faster than the "extremely slow motion" of 0.13 deg/Myr, to which we referred on line 112 (shown in Fig 3c, not 3b). Hence the estimate of Parés and Moore (2005) is hardly an "observation" in support of the slow rates of the Pacific plate.

Second, the estimates of hotspot and plate motion were derived by Parés and Moore (2005) only for the time after the formation of the bend (0-43 Ma in the original paper, or 0-47 Ma with the updated age of the bend). In contrast, our "extremely slow motion" was modelled for the time interval prior to the formation of the bend, 47-80 Ma (Fig. 3c), and we even spelled this out on lines 112-113 to avoid any confusion.

Nevertheless, we have modified the sentence from "but this would require an extremely slow rate of ~0.13^{o}/Myr, followed by a nearly six-fold acceleration (to 0.72^{o}/Myr) after 47 Ma. <u>No</u>

observations or models support such extremely slow motion of the Pacific plate during the 80-47 Ma interval" to "but this would require an extremely slow rate of ~0.13°/Myr, followed by a nearly six-fold acceleration (to 0.72°/Myr) after 47 Ma which is not supported by any observations or models". Thus we state that slow movements followed by a six-fold acceleration is not supported by observation/models rather than saying that the slow movement before 47 Ma is not supported by observation/models, although both statements are valid.
**Line 170**: Could such lower than expected latitudes be a hint of a rapid and recent drift to the north? Please provide some further explanation.
**RESPONSE:** Not really since even paleomagnetic data from the youngest rocks on Hawaii yield lower paleolatitudes. We have added a sentence on lines 178-180.
**Line 157**: the 0.4deg/Ma is the same order of magnitude as the one obtained using the productivity zone approach (Moore-Pares paper mentioned above), I think this is encouraging.
**RESPONSE:** Yes they argued for 0.32 cm/yr over the past 43 Ma and that the Hawaiian hotspot has had a similar velocity from the Cretaceous to recent times. We now mention this in lines 149-151, and the Parés & Moore paper has been added to the reference list (entry 29).<br>However, we should also point out that the Parés and Moore (2005) was for the last 53 Myr, whereas here we referred to the formation of the Emperor Seamounts (80-47 Ma).Also, their 32 mm/yr includes the dominantly eastward motion – note that the southward component of hotspot drift amounts to only 12 mm/yr and the direction is towards N114°E according to the Parés and Moore (2005) estimate.
**Figure 2:** Why not to describe the inset first (as this is the first prominent data that one sees), and then the X-Y plot? This is a key figure. Also, does it really help including the horizontal black line labeled "Hawaii Fixed"? I think it does not! Note that the Y-axis reads "… fixed Hawaii". Please consider deleting this line for simplicity, or else include in the caption the purpose of such line. Also I would refer to "six-pointed star" instead of David star (just an opinion).
**RESPONSE:** We have removed the inset diagram, simplified the diagram and revised the caption.
**Figure 3:** "… angular velocity to the NW".
**RESPONSE:** Ok added "to the north-west"
Otherwise this is a great contribution to the Earth Sciences community, and specifically to plate tectonics and beyond, and deserves to be published after considering the points brought up in this review.

Reviewer #3 (Remarks to the Author):

This is a valuable and timely review paper addressing an important issue: the extent to which the HEB is due to a change in the motion of the Pacific plate versus a change in the southward drift of the Hawaiian Hotspot. For a while it was generally accepted that both changes in Pacific plate motion and in the drift of the Hawaiian Hotspot were involved in the formation of the HEB. But several recent papers, e.g. by Wright et al. (2015) in Geology and Hassan et al. (2016) in Nature, have argued that the Bend is primarily due to changes in the drift of the Hawaiian Hotspot and that changes in Pacific plate motion were not important. I thought that Wilson's (2016) reply in Geology to Wright et al. (2015) really hit the nail on the head; he showed that there was indeed a very significant change in Pacific-Antarctic motion at 47 Ma at the latitude of the Hawaiian hotspot which Wright et a. (2015) had overlooked. I think that figure 5 from the paper under review here

also nails the importance of Pacific Absolute plate motion changes: take out Hassan et al.'s southward drift of the hotspot (Figure 5c) and you still have the Bend. Q.E.D.<br><br>This is not to say that this paper is fast reading. There are so many complex issues involved, that unless you are up on all of the various topics, parts of the paper are difficult to follow. The authors might want to consider moving more of the material to the Methods section. For example, it might be sufficient just to keep figure 6a in the paper and move the rest of figure 6 to the Methods section.
**RESPONSE:** We agree that may complex issues are discussed in the paper but we prefer to have all diagrams and text in the main paper and have now no supplementary information.
The title of this paper gets it right: the HEB is a mind-bending mystery. So, just to add to the list of mind-bending problems, I will point out that one issue that is given short shrift in this paper are the uncertainties in the plate circuit connecting the Indo-Atlantic plates to the Pacific plate. Because of the poorly constrained motion between East and West Antarctica prior to Chron 20, most studies these days looking at global plate motions, including this one, use the circuit from Ant-Aus-LHR-Pac first proposed by Steinberger et al. (2004). This gives an "improved fit" when linking Africa to Pacific hotspot trails. Great paper. However, the fact that this circuit gives an "improved fit" doesn't mean that it is the correct fit. In fact the plate motions between the LHR and the Pacific plate in the Eocene are not that well known. If the authors really take the LHR to be fixed to the Pacific plate between the cessation of spreading in the Tasman Sea at Chron 24 and the jump to using the East-West Antarctic-Pacific link at Chron 20 then that constrains there to be no motion between the Australia and Pacific plates in this time interval, i.e. making it one huge plate. Is that realistic? It would help to have some statement from the authors, perhaps after line 129, addressing the "unknown uncertainties" in the plate circuit and whether they might have any effect on the HEB mystery.
**RESPONSE:** We agree with most of the comments above and have extended the first paragraph in "Moving hotspot reference frames" (Lines 131-135).
A couple of typos:<br>e.g. **Line 115**: "a of change" should be "a change"
**RESPONSE:** Corrected

Reviewed by Steve Cande

Reviewers' Comments:

Reviewer #1:
Remarks to the Author:
I believe that the authors have adequately addressed my comments and concerns, and would be pleased to see the revised paper published.

Reviewer #2:
Remarks to the Author:
Congratulations for the paper, I am sure it will be of great interest for a broad audience in Earth Sciences. I appreciate your revisions, modifications and comments. All my comments are already made on the first review, and I acknowledge the improvement of the present version.

Reviewer #3:
Remarks to the Author:
The authors addition to lines 131 to 137 satisfactorily address my comments. I recommend publishing the revised version as is.
